# Systemic Inflammatory Response Index (SIRI) as a Predictive Marker for Adverse Outcomes in Children with New-Onset Type 1 Diabetes Mellitus

**DOI:** 10.3390/jcm13092582

**Published:** 2024-04-27

**Authors:** Alexandra-Cristina Scutca, Iulius Jugănaru, Delia-Maria Nicoară, Giorgiana-Flavia Brad, Meda-Ada Bugi, Raluca Asproniu, Lucian-Ioan Cristun, Otilia Mărginean

**Affiliations:** 1 Department XI Pediatrics, Discipline I Pediatrics, ‘Victor Babeş’ University of Medicine and Pharmacy of Timisoara, 300041 Timisoara, Romania; scutca.alexandra@umft.ro (A.-C.S.); nicoara.delia@umft.ro (D.-M.N.); brad.giorgiana@umft.ro (G.-F.B.); asproniu.raluca@umft.ro (R.A.); marginean.otilia@umft.ro (O.M.); 2 Department of Pediatrics I, Children’s Emergency Hospital “Louis Turcanu”, 300011 Timisoara, Romania; bugi.ada@umft.ro; 3 Research Center for Disturbances of Growth and Development in Children BELIVE, ‘Victor Babeş’ University of Medicine and Pharmacy of Timisoara, 300041 Timisoara, Romania; 4 Ph.D. School Department, ‘Victor Babeş’ University of Medicine and Pharmacy of Timisoara, 300041 Timisoara, Romania; lucian.cristun@umft.ro

**Keywords:** new-onset T1DM, systemic inflammatory response index (SIRI), acute complications, prolonged ICU LOS

## Abstract

(1) **Background**: Although most cases of new-onset type 1 diabetes mellitus (T1DM) are managed without serious events, life-threatening complications do arise in a subset of patients. Our objective was to assess the correlation between elevated SIRI values and adverse events related to the onset of T1DM. (2) **Methods:** This retrospective study, spanning ten years, included 187 patients with new-onset T1DM divided into three groups based on SIRI tertiles. The primary outcome was the occurrence of acute complications during hospital admission, while the secondary outcome was prolonged Intensive Care Unit (ICU) admission. (3) **Results:** Patients with high SIRI values were more likely to experience higher disease activity, leading to longer ICU admission times and more frequent complications. Multivariate logistic regression analysis revealed that the SIRI was independently associated with acute complications (*p* = 0.003) and prolonged ICU length of stay (*p* = 0.003). Furthermore, receiver operating characteristic analysis demonstrated the SIRI’s superior predictive accuracy compared to venous pH (AUC = 0.837 and AUC = 0.811, respectively) and to the individual component cell lineages of the SIRI. (4) **Conclusions:** These findings emphasize the potential utility of the SIRI as a prognostic marker in identifying patients at increased risk during T1DM hospital admissions.

## 1. Introduction

Type 1 diabetes mellitus (T1DM), an immune-mediated condition, is one of the most common chronic diseases in children [1]. It culminates in the complete or partial loss of pancreatic β cells, leading to reduced production of endogenous insulin and necessitating exogenous insulin administration to sustain proper energy production [2]. DKA, a frequent form of presentation in new-onset T1DM [3], is the leading cause of morbidity and mortality in children, mainly due to its potential association with acute complications [4]. Among these complications, the development of cerebral edema stands out as the most concerning outcome, with reported incidences of up to 1% among pediatric DKA patients [5]. Notably, it carries high mortality rates of 20–40% [6], along with the potential for long-term neurologic sequelae [6,7,8]. Other acute complications that may arise in the presence of DKA include acute kidney injury [AKI], electrolyte imbalances that can lead to arrhythmias, acute pancreatitis, and deep venous thrombosis [9,10,11,12].

Inflammation plays a pivotal role in the initiation and progression of chronic diseases [13,14,15,16,17]. Recent studies on T1DM have unveiled a broader role for innate immunity than previously assumed. Particularly, inflammation localized within the pancreatic islets, known as insulitis, contributes to a gradual decline in insulin-producing β cells [18,19,20]. However, the direct quantification of inflammatory cell infiltration linked with T1DM faces limitations due to its invasive nature. Given that exploring the distribution of peripheral white blood cells can offer valuable insights into the inflammatory process, assessing indices derived from complete blood count (CBC) parameters as markers of inflammatory conditions appears reasonable [21]. A growing body of research focuses on CBC-derived indices in chronic diseases, including diabetes. Among these, the most evaluated index in diabetic patients has been the neutrophil-to-lymphocyte ratio (NLR) [22,23,24,25]. However, while much of the research has centered on the role of neutrophils and lymphocytes, monocytes also appear to exert a notable impact on the pathogenesis of T1DM [26,27,28,29] and its complications, such as cerebral edema and AKI [30,31]. Consequently, investigating an index that integrates monocytes seems useful. The Systemic Inflammatory Response Index (SIRI), a composite index incorporating information from NLR components and monocytes [32], could thus offer pertinent insights into the cellular response in T1DM. Nevertheless, studies on the use of the SIRI in T1DM remain scarce.

In this context, our aim was to examine the association between the SIRI, as a potential inflammatory marker, and adverse events occurring during the onset of T1DM in children.

## 2. Materials and Methods

### 2.1. Study Design and Protocol

This retrospective single-center cross-sectional study was performed at a tertiary referral center specializing in pediatric diabetes in Romania. We reviewed the medical charts of 219 patients diagnosed with T1DM in the Pediatric Emergency Hospital ‘Louis Turcanu’ from Timisoara, Romania, between 1 January 2014 and 31 December 2023. The study protocol, conducted with Good Clinical Practice (Declaration of Helsinki from 1975, revised in 2013), was approved by the hospital’s Institutional Review Board (protocol no. 14571/17.11.2023). Informed consent was waived in compliance with ethical regulations due to the retrospective nature of this study. The inclusion criteria comprised individuals who were (1) under 18 years of age and (2) diagnosed with new-onset T1DM based on the 2021 criteria of the American Diabetes Association (ADA) [33]. Patients with (1) preexisting medical conditions known to modify hematological parameters, such as hematologic or autoimmune diseases, (2) those receiving chronic corticotherapy, and (3) those with incomplete medical data were excluded from the study. DKA was considered if patients exhibited plasma glucose levels > 11 mmol/L, urine ketone levels categorized as moderate to high (+ to +++), and arterial pH values < 7.30 upon admission [34]. The severity classification of DKA followed the guidelines outlined by the American Diabetes Association: mild DKA was defined as 7.20 ≤ pH < 7.30, moderate DKA as 7.10 ≤ pH < 7.20, and severe DKA as pH < 7.10 [34]. Overt cerebral edema was diagnosed on clinical basis in patients presenting with a Glasgow Coma Scale (GCS) score < 14 and sudden alterations in neurological status, including altered levels of consciousness (confusion, lethargy, or coma) or signs of increased intracranial pressure (severe headache, recurrent vomiting, hypertension, seizures, or papilledema), necessitating administration of hypertonic solution or intubation [35]. Acute kidney injury was characterized based on the serum creatinine criteria outlined by the Kidney Disease/Improving Global Outcomes guidelines. AKI stages—no AKI, stage 1, stage 2, or stage 3—were determined based on serum creatinine values < 1.5, 1.5 to <2, 2 to <3, and ≥3 times the basal serum creatinine, respectively [36]. Due to the absence of baseline creatinine data prior to admission, we derived the expected baseline creatinine (EBC) levels by utilizing an estimated glomerular filtration rate (eGFR) [37,38] of 120 mL/min/1.73 m^2^ and patients’ body height (calculated using the Schwartz formula) [39]. Severe electrolyte imbalances were diagnosed based on the following cut-off values: plasma sodium < 120 mEq/L for severe hyponatremia, plasma sodium > 150 mEq/L for severe hypernatremia, plasma potassium < 2.5 mEq/L for hypokalemia, and plasma potassium > 6.5 mEq/L for hyperkalemia. Deep vein thrombosis (DVT) was diagnosed through clinical assessment, imaging studies such as Doppler ultrasound, and laboratory tests for D-dimer levels. Acute pancreatitis was diagnosed based on clinical symptoms, a CT scan showing pancreatic inflammation, and elevated levels of serum amylase and lipase. Prolonged Intensive Care Unit length of stay (ICU LOS) was considered >48 h of ICU-level care [40]. Co-infection was defined as the presence of acute infections detected upon hospital admission, including upper and lower respiratory tract infections, digestive infections, and urinary tract infections. Sepsis was characterized by suspected or confirmed infection meeting at least two of the systemic inflammatory response syndrome (SIRS) criteria: (1) body temperature > 38.5 °C or <36 °C, (2) mean heart rate exceeding +2 standard deviations (SDs) above the normal range for age in the absence of external stimuli, or unexplained persistent elevation [41].

### 2.2. Clinical and Laboratory Data

Patient characteristics, encompassing age, gender, physical examination findings upon admission, GCS score upon admission, co-infection status, and DKA status, were collected. The length of stay in the Intensive Care Unit was determined, along with documentation of critical care interventions, notably the use of mechanical ventilation. Laboratory parameters collected upon admission comprised a CBC, analyzed using a Sysmex XN-550 automated hematology analyzer (Sysmex Corporation, Kobe, Japan), and several biochemistry tests. The latter included C-reactive protein (CRP), fasting glucose level, blood urea nitrogen (BUN), and creatinine, which were assessed using an automatic analyzer (Hitachi 747, Hitachi, Tokyo, Japan). Glycated hemoglobin (HbA1c) was measured via high-performance liquid chromatography (Cobas E 411-Roche, Japan, Tokyo). Additionally, C-peptide was evaluated using an automated chemiluminescent assay (Cobas E 411-Roche, Tokyo, Japan). The SIRI score was calculated based on peripheral blood cell counts using the following formula: (absolute neutrophil count × absolute monocyte count)/absolute lymphocyte count [42].

### 2.3. Key Outcome Measures

The primary outcome of interest was the occurrence of acute complications during hospitalization for T1DM, specifically overt cerebral edema, acute kidney injury, severe electrolyte imbalances, acute pancreatitis, and deep vein thrombosis. This study aimed to assess the predictive role of the SIRI specifically in relation to these complications. The secondary outcome measure was the need for ICU care exceeding 48 h.

### 2.4. Use of OpenAI ChatGPT-3.5

The ChatGPT-3.5 model, developed by OpenAI [43], was used for language and grammar checks throughout the article. The authors conducted thorough reviews, edits, and revisions of the ChatGPT-generated texts, assuming accountability for the content of the publication.

### 2.5. Statistical Analysis

Statistical analysis was performed with the help of Statistical Package for Social Sciences software (SPSS v28.0.1.1. Armonk, IBM Corp, Armonk, NY, USA). Patients were divided into three study groups based on SIRI tertiles and characterized using descriptive statistics, including percentages, medians, and interquartile ranges. The normality of data distribution was verified using the Shapiro–Wilk test. Numerical variables, displaying non-normal distribution, were computed as median and interquartile range (IQR), and the Kruskal–Wallis test was used for intergroup comparison. Categorical variables were displayed as numerical and percentile values, with intergroup comparisons performed using the Chi-squared test. Spearman’s rank correlation coefficient (ρ) was used to determine relationships between clinical parameters and adverse events such as acute complications, prolonged ICU LOS, and the need for mechanical ventilation. Additionally, multivariate logistic regression analysis explored independent factors associated with acute complications and prolonged ICU LOS. Finally, receiver operating characteristic (ROC) curve analysis was employed to further elucidate the prognostic accuracy of the SIRI in identifying patients with acute complications. Youden’s index (sensitivity + specificity − 1) determined appropriate cut-off values. A two-tailed *p*-value < 0.05 was considered statistically significant.

## 3. Results

### 3.1. Baseline Characteristics of the Study Population

Data from 186 children aged 1 to 18 years diagnosed with T1DM were included in this study. Patients were categorized into three groups based on SIRI tertiles. Differences in demographic, clinical, and laboratory characteristics of the subjects are illustrated in Table 1. The study population had a median age of 9.4 years (IQR: 5.1–12.7 years). Gender distribution did not reveal significant differences across study groups (*p* = 0.267). As shown in Table 1, patients with high SIRI values were more likely to be female and to experience higher disease activity. This corresponded to longer durations of ICU admission, increased occurrence of complications, and an increased need for mechanical ventilation. Regarding laboratory parameters, patients with high SIRI values exhibited a significant increase in WBCs, neutrophils, monocytes, platelets, and CRP, along with a significant decrease in lymphocytes and eosinophils. Furthermore, patients with high SIRI values were more likely to have lower venous pH and C-peptide. HbA1c values were similar across all SIRI tertiles.

### 3.2. Comparison of SIRI According to Infection Status in Severe DKA Patients

**Figure 1:**
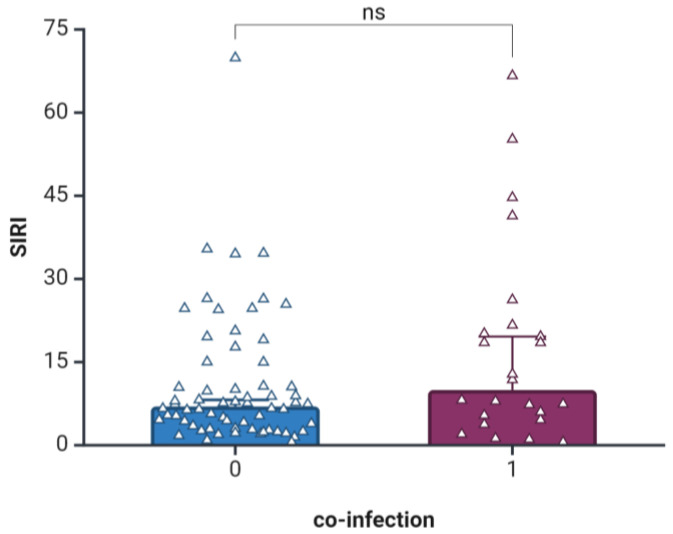
Boxplot diagram of Systemic Inflammatory Response Index (SIRI) according to co-infection status. 0, no co-infection; 1, co-infection; ns, not significant (*p* = 0.185). Transparent triangles represent individual values. Created with Biorender.com accessed on 12 March 2024.

When stratifying patients according to co-infection status, we observed no statistically significant differences between the median SIRI values of patients with co-infection (7, IQR: 3.36–11.73) upon admission and those without co-infection (9.96, IQR: 5.18–20.51), as seen in Figure 1.

### 3.3. Correlation Analysis of SIRI with Adverse Events

**Figure 2:**
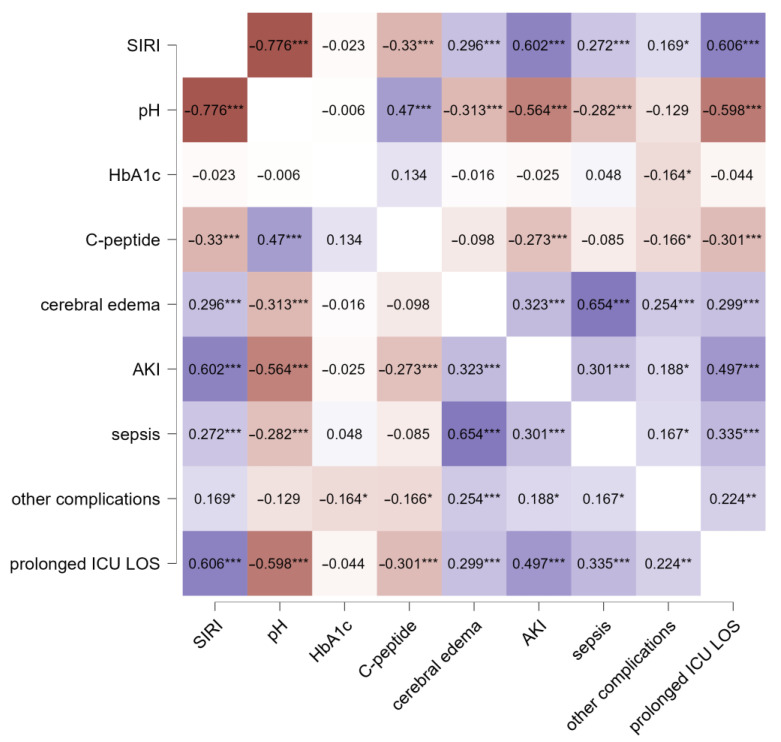
Correlation analysis of median SIRI values, glucose metabolism markers, and adverse events. Abbreviations: SIRI, Systemic Inflammatory Response Index; HbA1c, glycated hemoglobin A1c; AKI, acute kidney injury; ICU, Intensive Care Unit; LOS, length of stay. The range of colors in the figure reflects the strength of significance, with deeper shades indicating stronger associations and lighter tones suggesting comparatively lower statistical significance. Positive correlation coefficients indicate positive associations, while negative correlation coefficients signify negative associations. Significance levels are as follows: *** *p* < 0.001, ** *p* < 0.01, * *p* < 0.05.

The relationship between median SIRI values and adverse events occurring during hospital admission for T1DM onset (Figure 2) was evaluated using Spearman correlation analysis. The most notable correlations between the SIRI and adverse events during hospital stay were observed with prolonged ICU LOS (*ρ* = 0.606) and AKI (*ρ* = 0.602). Additionally, mean SIRI values significantly correlated with overt cerebral edema (*ρ =* 0.296) and sepsis (*ρ =* 0.272). Similar correlations were observed for venous pH. When considering glucose metabolism markers, only C-peptide significantly correlated with unfavorable events during the hospital stay, although to a lesser extent than the SIRI and venous pH (Figure 2).

### 3.4. Association Analysis between SIRI and Adverse Events

Furthermore, we employed multivariate logistic regression to explore the relationship between the SIRI and adverse events, including acute complications and ICU LOS exceeding 48 h, both in the entire study population as well as only in severe DKA patients (Table 2). We employed a stepwise selection approach to mitigate the risk of overfitting resulting from the inclusion of numerous assessed variables. This method automatically identified a subset of predictors considered the most influential in the model. When analyzing the entire study population, the SIRI and venous pH were retained in the final model, demonstrating statistical significance. After adjusting for multiple confounding factors, the SIRI retained statistical significance as an independent factor associated with acute complications (*p* = 0.003). Additionally, in logistic regression modeling for the outcome of prolonged ICU LOS, the SIRI remained significant as an independent predictor (*p* < 0.001).

In addition, we conducted an analysis focusing on a subset of patients, specifically those with severe DKA, to determine whether the SIRI maintains statistical significance as an independent factor associated with adverse events. Multivariate logistic regression, employing a stepwise selection approach, demonstrated that the SIRI remained significantly associated with acute complications and prolonged ICU LOS, even when specifically examining patients with severe DKA.

#### ROC Analysis Regarding the Predictive Accuracy of SIRI for Adverse Events

**Figure 3:**
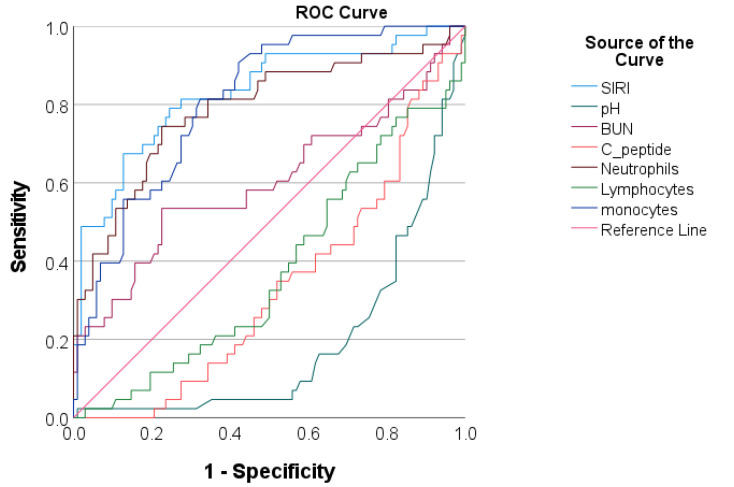
Receiver operating characteristic curve analysis for evaluating the performance of inflammatory and metabolic markers in discriminating acute complications.

**Figure 4:**
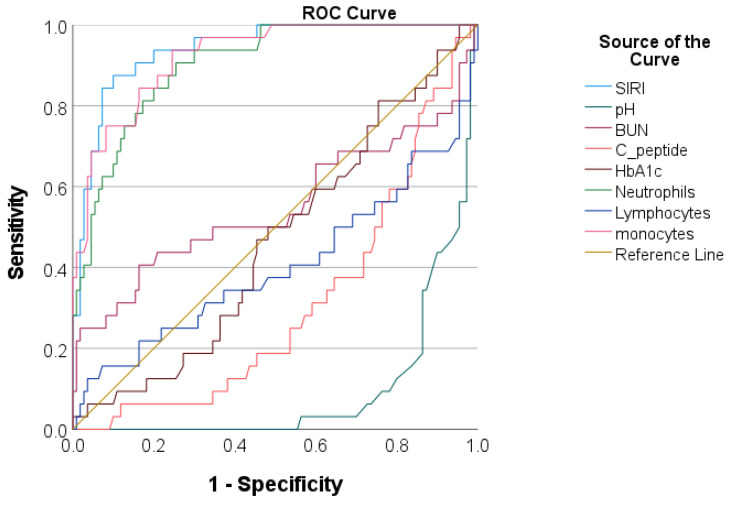
Receiver operating characteristic curve analysis for evaluating the performance of inflammatory and metabolic markers in discriminating prolonged ICU LOS.

We evaluated the predictive accuracy of the SIRI regarding both acute complications and prolonged ICU LOS and compared it with venous pH and glucose metabolism markers. The area under the curve was computed, identifying optimal cut-off values. As depicted in Table 3 and Table 4 and Figure 3 and Figure 4, the SIRI demonstrated excellent accuracy for both acute complications (AUC = 0.837, with 82.0% sensitivity and 72.0% specificity) and prolonged ICU LOS (AUC = 0.900, with 89.6% sensitivity and 73.9% specificity). We also assessed the predictive accuracy of each cell lineage within the hematological index, with the SIRI demonstrating superior performance compared to its components.

## 4. Discussion

In a retrospective data analysis spanning ten years of patients with new-onset T1DM, we observed a significant association between higher SIRI values and adverse events during hospital admission. This association can be attributed to the severity of DKA, which often leads to various complications and results in alterations in the CBC profile of patients [44]. Some of these alterations are reflected in the SIRI calculation. Although the exact processes driving the progression of DKA remain unclear, multiple studies indicate that inflammation may play a significant role in its advancement [45,46,47,48]. Unlike earlier literature, which emphasized the dysregulation of adaptive immunity, recent studies have highlighted the importance of innate immunity in the immunopathology of T1DM [49]. In particular, there is an emphasis on the involvement of innate immunity, both in the initial immune attack on pancreatic β cells and in subsequent stages, when it contributes to the stabilization and perpetuation of insulitis [17,50,51,52,53,54]. In addition to cytokines, inflammation also triggers cellular responses, leading to changes across multiple cell lineages within the hematopoietic system [55]. During the last decade, several CBC-derived indices have been investigated as potential inflammatory biomarkers, with the neutrophil-to-lymphocyte ratio [56] being the prototype among them. In their study, Cheng et al. found that the NLR could indicate the underlying severity of acute systemic inflammation in adult DKA patients, particularly those without infection [22]. Subsequently, this association was also demonstrated in pediatric patients experiencing new-onset DKA [57]. However, in addition to the alteration in absolute neutrophil and lymphocyte count in T1DM, studies have also described an increase in circulating monocytes [28]. During inflammatory conditions, human peripheral monocytes serve as antigen-presenting cells to activate T cells [58,59] and produce cytokines that influence T-cell differentiation [60]. Following antigen stimulation, intermediate monocytes assume the primary role in generating inflammatory factors, such as interleukin (IL)-1α, IL-6, and TNF-α [61]. These TNF-α levels correlate with the severity of T1DM [62,63,64]. Ren et al. observed a correlation between a higher population of intermediate monocytes and increased memory T cells in children with recent-onset T1DM. These memory T cells secreted high levels of IL-2 and IFN-γ [65]. Given the role of monocytes in diabetes, a CBC-derived index incorporating both NLR and monocyte information, the SIRI, has been investigated in several adult studies concerning diabetes complications, particularly in type 2 diabetes. Wang et al. suggested the SIRI as an independent risk factor for diabetic retinopathy [66]. Lin et al. associated it with the risk of cardiovascular disease [67], while Zhang et al. linked it to cardio-cerebrovascular disease mortality among adults with type 2 DM [68]. However, studies addressing its use in children with T1DM are notably limited.

To the best of our knowledge, the present study is the first to investigate the potential utility of using the SIRI in children with new-onset T1DM. Our study population had a median age of 9.4 years, similar to findings from previous studies [69,70], although slightly older than the average age reported by Boboc et al. [71]. Female patients had a marginal prevalence (54.3%), especially among patients from the high tertile, although this difference did not reach statistical significance. Among patients presenting with DKA, most (64.7%) had severe DKA. The incidence of severe DKA is higher than that reported in some previous studies [3,72]. This might be attributed to the fact that, as a tertiary reference center with a pediatric ICU facility, our hospital primarily handles severe cases, drawing patients from most of the West of the country. Conversely, patients who can be initiated on subcutaneous insulin are typically managed at other healthcare facilities within their respective areas. The occurrence of adverse events during hospitalization included 56 cases of AKI, 8 cases of overt cerebral edema, 13 patients with severe electrolyte imbalance, 1 instance of deep vein thrombosis, and 1 case of acute pancreatitis. No deaths occurred within the entire study population, and none of the patients with AKI required dialysis. Most of the cases with overt cerebral edema presented with sepsis at the moment of admission. This is consistent with a previous study that disclosed the co-occurrence of infections in children with cerebral edema [73]. Furthermore, concerning the length of stay in the ICU, the median duration for the entire study population was 8 h. Among patients with DKA, the median ICU LOS was 38 h, rising to 48 h for those with severe DKA. Previous studies have reported a slightly higher LOS of 48–60 h regarding children with DKA [74,75].

In order to analyze the relationship between the SIRI and adverse events during hospitalization, we divided the study population into low-, moderate-, and high-SIRI tertiles. Significant discrepancies were observed in the clinical and laboratory profiles of the three groups of patients, as well as in their hospital course. These observations suggest a relationship between the increase in SIRI values and the progression of disease severity, leading to acute complications. To further characterize this relationship, we applied additional analytical tests. Our results indicated a positive correlation between the SIRI and individual acute complications, as well as with prolonged ICU LOS, similar to the pattern observed with venous pH. Furthermore, the results of multivariate logistic regression showed that higher median SIRI values were independently associated with both acute complications and prolonged ICU LOS, even after adjusting for various potential confounders such as co-infection and venous pH. Additionally, the SIRI demonstrated excellent predictive capability in ROC analysis in identifying patients with acute complications. At a cut-off value of 2.39, it demonstrated a sensitivity of 83.3% and a specificity of 70.8%, slightly surpassing those of venous pH. Finally, we also noted that the predictive accuracy of the SIRI surpassed that of its component cell lineages. These results imply that the collective data offered by the SIRI may provide a more thorough evaluation of the inflammatory status and disease severity compared to its components in children with T1DM.

The results of the present study must be interpreted in light of several limitations. First, this study’s retrospective single-center design presents concerns regarding potential selection bias, mainly due to its location in a diabetes referral center, which could have led to an overrepresentation of severe cases. Furthermore, incomplete documentation for patients referred from territorial hospitals necessitated their exclusion from the analysis, further aggravating the potential for bias. Second, the SIRI was assessed only at one time point. Serial measurements of the SIRI and its changes in children with new-onset T1DM could offer a more comprehensive picture of the dynamic correlation between them. Concerning the study population, the relatively modest patient sample size could have influenced this study’s statistical power. Additionally, since it consisted of real-life patients, several of them had acute infections, which are known to influence CBC parameters. However, we included patients with co-infections, as they represented one in five children and offered a more realistic and complete picture of pediatric T1DM patients. There were no significant statistical differences in SIRI values when comparing children with and without infections. As such, our results can be viewed as exploratory. In the future, improving the experimental design could involve adopting a prospective approach with longitudinal studies across multiple centers, ensuring comprehensive documentation of patient data. These improvements might be beneficial for validating our findings.

## 5. Conclusions

In summary, this study revealed that individuals with high SIRI values faced an increased risk of adverse events during hospital admission for new-onset T1DM. This offers a fresh perspective on risk assessment, potentially leading to early intervention strategies by introducing the SIRI as a predictive marker. Furthermore, this practical tool allows clinicians in minimally equipped healthcare facilities to identify high-risk pediatric patients requiring admission to ICU-equipped medical facilities. Patients initially presenting in ambulatory settings with minimal routine investigations, displaying clinical signs of diabetes and elevated glucose and SIRI levels, would benefit from expedited referral to an ICU-equipped hospital. While our findings do not substitute traditional diagnostic criteria, this study provides complementary laboratory data regarding this subset of patients. However, due to the single-center design of this study and its conduct in a tertiary reference center, caution should be exercised in extrapolating the results to the general population.

## Figures and Tables

**Table 1:** Baseline clinical and laboratory parameters stratified by SIRI tertiles.

Variables	Tertiles of SIRI			*p*-Value
	1 (0.14–1.07) *n* = 62	2 (1.19–5.04) *n* = 62	3 (5.31–69.88) *n* = 62	
Demographic characteristics				
Age (years)	9.05 (4.07, 12.7)	8.10 (4.75, 12.2)	10.8 (7.05, 14)	0.086
Females	46.8 (29)	54.8 (34)	61.3 (38)	0.267
Height (cm)	134 (105–158)	127 (109–153)	143 (122–156)	0.192
Weight (kg)	28.5 (16.6–44)	25 (15.7–44)	33.5 (19–47.7)	0.213
BMI (kg/m^2^)	15.6 (14.3–18.06)	15.9 (13.8–18.1)	16.1 (13.9–19.9)	0.695
Co-infection	11.3 (7)	16.1 (10)	32.3 (20)	**0.011**
DKA status:				
no DKA	56.5 (35)	24.2 (15)	0 (0)	**<0.001**
mild DKA	22.6 (14)	11.3 (7)	0 (0)	**<0.001**
moderate DKA	12.9 (8)	24.2 (15)	6.5 (4)	**<0.001**
severe DKA	8.1 (5)	40.3 (25)	93.5 (58)	**<0.001**
GCS	15 (15, 15)	15 (15, 15)	14 (12, 15)	**0.001**
LOS in ICU (days)	0 (0, 0)	0 (0, 39)	55.5 (38, 73)	**0.001**
Acute complications:				
Cerebral edema	0 (0)	0 (0)	12.9 (8)	**<0.001**
AKI	3.2 (2)	19.4 (12)	67.7 (42)	**0.003**
Sepsis	1.6 (1)	0 (0, 0)	11.3 (7)	**<0.001**
Other (severe electrolyte imbalance, DVT, acute pancreatitis)	3.2 (2)	4.8 (3)	12.9 (8)	**0.003**
Mechanical ventilation	1.6 (1)	0 (0)	9.7 (6)	**0.002**
Death	0 (0)	0 (0)	0 (0)	**-**
Laboratory parameters				
WBCs (×10^3^/mm^3^)	7.8 (6.29, 10.08)	11.43 (8.66, 15.08)	18.91 (15.98, 25.76)	**0.001**
Neutrophils (×10^3^/mm^3^)	3.63 (2.86, 4.53)	7.27 (5.27, 9.46)	14.68 (11.87, 23.98)	**0.001**
Lymphocytes (×10^3^/mm^3^)	3.43 (2.43, 4.67)	2.89 (2.06, 4.01)	2.40 (1.70, 3.44)	**<0.001**
Monocytes (×10^3^/mm^3^)	0.56 (0.41, 0.71)	0.90 (0.68, 1.25)	2.01 (1.38, 2.81)	**0.001**
Eosinophils (×10^3^/mm^3^)	0.10 (0.05, 0.19)	0.05 (0.02, 0.14)	0.01 (0, 0.02)	**<0.001**
Platelets (×10^3^/mm^3^)	283 (188, 342)	319 (255, 400)	363 (298, 438)	**<0.001**
Venous pH	7.31 (7.23, 7.36)	7.14 (7.01, 7.28)	6.95 (6.88, 7.02)	**0.001**
Glycemia (mg/dL)	347 (269, 463)	430 (344, 500)	471 (379, 560)	**<0.001**
HbA1c (%)	11.70 (9.95, 13.24)	11.30 (10.15, 12.72)	11.53 (10.20, 12.69)	0.871
C-peptide (nmol/L)	0.572 (0.369, 0.726)	0.542 (0.232, 0.739)	0.310 (0.202, 0.479)	**<0.001**
C-reactive protein (mg/L)	0.61 (0.21–1.21)	1.10 (0.83–3.85)	3.66 (1.34–10.49)	**<0.001**
BUN (mmol/L)	3.49 (2.93, 4.29)	3.57 (2.66, 4.31)	4.35 (2.69, 6.50)	**0.048**
Creatinine (umol/L)	43 (34, 50)	46 (37, 60)	61 (47, 78.2)	**<0.001**
ALT (U/L)	12 (9–15)	12 (10–15)	13 (10–18)	0.575
AST (U/L)	16.5 (13–20)	14.5 (12–20)	14 (10–23)	0.283

Data are expressed as median (IQR) or percentage (*n*, %). SIRI, Systemic Inflammatory Response Index; DKA, diabetic ketoacidosis; GCS, Glasgow Coma Scale; LOS, length of stay; ICU, Intensive Care Unit; AKI, acute kidney injury; DVT, deep vein thrombosis; WBCs, white blood cells; HbA1c, glycated hemoglobin A1c; CRP, C-reactive protein; BUN, blood urea nitrogen; ALT, alanine aminotransferase; AST, aspartate aminotransferase. Statistically significant differences, indicating a probability value of *p* < 0.05, are highlighted in bold.

**Table 2:** Association between SIRI and adverse events.

	Non-Adjusted ^a^		Model 1 ^b^		Model 2 ^c^	
	OR (95%CIs)	*p*-Value	OR (95%CIs)	*p*-Value	OR (95%CIs)	*p*-Value
Entire study population						
Acute complications	1.180 (1.106–1.259)	**<0.001**	1.193 (1.116–1.276)	**<0.001**	1.103 (1.035–1.176)	**0.003**
Prolonged ICU LOS	1.218 (1.135–1.307)	**<0.001**	1.250 (1.157–1.350)	**<0.001**	1.154 (1.064–1.252)	**<0.001**
Severe DKA						
Acute complications	1.091 (1.027–1.158)	**0.004**	1.103 (1.034–1.177)	**0.003**	1.095 (1.031–1.164)	**0.003**
Prolonged ICU LOS	1.047 (1.010–1.086)	**0.012**	1.058 (1.015–1.103)	**0.008**	1.053 (1.012–1.095)	**0.010**

^a^ Non-adjusted model adjusted for nothing; ^b^ model 1 adjusted for age, gender; ^c^ model 2 adjusted for age, gender, co-infection, venous pH, HbA1c. OR, odds ratio; CI, confidence interval; ICU, Inensive Care Unit; LOS, length of stay. Statistically significant differences, indicating a probability value of *p* < 0.05, are highlighted in bold.

**Table 3:** Comparison of inflammatory and metabolic markers in discriminating acute complications.

	AUC	SE	95%CI	Sensitivity	Specificity	Cut-Off	*p*-Value
SIRI	0.837	0.032	0.774–0.900	0.820	0.720	2.66	**0.001**
pH	0.811	0.032	0.748–0.874	0.800	0.607	7.01	**0.001**
BUN	0.586	0.050	0.488–0.685	0.576	0.549	3.75	0.064
C-peptide	0.682	0.045	0.595–0.769	0.673	0.568	0.374	**0.001**
HbA1c	0.463	0.045	0.374–0.551	0.463	0.508	11.4	0.431
Neutrophils	0.793	0.036	0.722–0.864	0.787	0.720	8.21	**0.001**
Lymphocytes	0.584	0.044	0.498–0.670	0.576	0.525	2.68	0.063
Monocytes	0.812	0.032	0.749–0.874	0.803	0.664	0.935	**0.001**

Abbreviation: SIRI, Systemic Inflammatory Response Index; HbA1c, glycated hemoglobin A1c; BUN, blood urea nitrogen. Statistically significant differences, indicating a probability value of *p* < 0.05, are highlighted in bold.

**Table 4:** Comparison of inflammatory and metabolic markers in discriminating prolonged ICU LOS.

	AUC	SE	95%CI	Sensitivity	Specificity	Cut-Off	*p*-Value
SIRI	0.900	0.023	0.855–0.945	0.896	0.739	3.24	**<0.001**
pH	0.894	0.023	0.849–0.940	0.877	0.625	6.97	**0.001**
BUN	0.543	0.059	0.427–0.658	0.522	0.627	4.09	0.393
C-peptide	0.709	0.047	0.617–0.802	0.708	0.647	0.367	**0.001**
HbA1c	0.471	0.051	0.371–0.571	0.455	0.523	11.5	0.559
Neutrophils	0.865	0.028	0.810–0.919	0.854	0.703	8.32	**0.001**
Lymphocytes	0.557	0.053	0.454–0.660	0.551	0.583	2.83	0.239
Monocytes	0.882	0026	0.831–0.933	0.875	0.717	1.14	**0.001**

Abbreviation: SIRI, Systemic Inflammatory Response Index; HbA1c, glycated hemoglobin A1c; BUN, blood urea nitrogen. Statistically significant differences, indicating a probability value of *p* < 0.05, are highlighted in bold.

## Data Availability

Data can be made available upon reasonable request due to ethical restrictions.
